# Predicting Readmission Among High-Risk Discharged Patients Using a Machine Learning Model With Nursing Data: Retrospective Study

**DOI:** 10.2196/56671

**Published:** 2025-03-05

**Authors:** Eui Geum Oh, Sunyoung Oh, Seunghyeon Cho, Mir Moon

**Affiliations:** 1 College of Nursing, Yonsei University, Seoul, Republic of Korea; 2 Mo-Im Kim Nursing Research Institute, Yonsei University, Seoul, Republic of Korea; 3 Institute for Innovation in Digital Healthcare, Yonsei University, Seoul, Republic of Korea; 4 School of Nursing, Yale University, New Haven, CT, United States; 5 Digital & Technology Group, CJ CheilJedang, Suwon, Republic of Korea; 6 Department of Nursing, Graduate School, Yonsei University, Seoul, Republic of Korea; 7 Department of Nursing, Daejeon University, 62, Daehak-ro, Dong-gu, Daejeon, 34520, Republic of Korea, 82 10-9973-8813

**Keywords:** machine learning, EHR, electronic health record, electronic medical record, EMR, artificial intelligence, readmission, nursing data, clinical decision support, prediction, predictive, discharge, admission, hospitalization

## Abstract

**Background:**

Unplanned readmissions increase unnecessary health care costs and reduce the quality of care. It is essential to plan the discharge care from the beginning of hospitalization to reduce the risk of readmission. Machine learning–based readmission prediction models can support patients’ preemptive discharge care services with improved predictive power.

**Objective:**

This study aimed to develop a readmission early prediction model utilizing nursing data for high-risk discharge patients.

**Methods:**

This retrospective study included the electronic medical records of 12,977 patients with 1 of the top 6 high-risk readmission diseases at a tertiary hospital in Seoul from January 2018 to January 2020. We used demographic, clinical, and nursing data to construct a prediction model. We constructed unplanned readmission prediction models by dividing them into Model 1 and Model 2. Model 1 used early hospitalization data (up to 1 day after admission), and Model 2 used all the data. To improve the performance of the machine learning method, we performed 5-fold cross-validation and utilized adaptive synthetic sampling to address data imbalance. The 6 algorithms of logistic regression, random forest, decision tree, XGBoost, CatBoost, and multiperceptron layer were employed to develop predictive models. The analysis was conducted using Python Language Reference, version 3.11.3. (Python Software Foundation).

**Results:**

In Model 1, among the 6 prediction model algorithms, the random forest model had the best result, with an area under the receiver operating characteristic (AUROC) curve of 0.62. In Model 2, the CatBoost model had the best result, with an AUROC of 0.64. BMI, systolic blood pressure, and age consistently emerged as the most significant predictors of readmission risk across Models 1 and 2. Model 1, which enabled early readmission prediction, showed a higher proportion of nursing data variables among its important predictors compared to Model 2.

**Conclusions:**

Machine learning–based readmission prediction models utilizing nursing data provide basic data for evidence-based clinical decision support for high-risk discharge patients with complex conditions and facilitate early intervention. By integrating nursing data containing diverse patient information, these models can provide more comprehensive risk assessment and improve patient outcomes.

## Introduction

Readmission is an unintended outcome that occurs in patients discharged from the hospital. In South Korea, the 30-day readmission rate in tertiary general hospitals in 2020 was approximately 30%, increasing yearly along with readmission cost statistics [1]. According to a Center for Medicare and Medicaid Services report in the United States, readmission rates for patients reach 2 million yearly, with readmissions costing $26 billion [2]. Despite the growing interest and literature on readmissions, unplanned readmission rates still need to be managed.

It is essential to accurately predict and select high-risk discharge patients to reduce readmission costs and improve the quality of medical care. Predictive modeling is an efficient way to stratify patient readmission risk and optimize the allocation of clinical resources by providing preventive interventions to high-risk patients [3]. Traditional statistical methods for creating predictive models have focused on inference, which involves creating a mathematical model of a data-generating process to formalize an understanding of how it works or to test hypotheses. The problem is that as the sample size of the data grows and the number of input variables increases, the possible associations between variables increase, making the model more complex and the statistical inference less accurate [4]. In particular, traditional risk prediction models, such as regression analysis, show limited predictive power [5]. In response to these challenges, machine learning methodologies have emerged as a powerful alternative. These approaches employ diverse algorithms to systematically process complex and voluminous datasets, identifying patterns and generating predictions. This capability proves particularly advantageous when dealing with “big data” characterized by numerous input variables and complex nonlinear interactions [6]. However, it is crucial to note that the application of machine learning in medical contexts presents certain challenges. In the absence of robust predictors and comprehensive domain-specific knowledge, establishing direct correlations between machine learning outcomes and existing medical information can be problematic. Despite these limitations, recent advancements have led to the development of machine learning–based high-prediction models. These models function as advanced predictive analysis technologies, capable of identifying and processing diverse patient information and conditions. Through the analysis of high-dimensional medical data, these sophisticated models generate hierarchical predictive frameworks with enhanced prognostic capabilities. This approach effectively addresses the inherent limitations of conventional statistical methods, thereby facilitating the implementation of personalized treatment strategies tailored to individual patients [7].

Treatment of high-risk discharged patients with high readmission is focused only at discharge, mainly by providing postdischarge care interventions [8]. Additionally, most existing readmission prediction models only utilize medical information data such as the patient’s diagnostic tests [9]. Although nursing data in the early stages of a patient’s hospitalization include comprehensive and direct information on physical and functional health factors, psychosocial characteristics, and socioeconomic status, studies have yet to be conducted on developing predictive models utilizing the usefulness of this nursing assessment information [10]. Predicting a patient’s readmission should be done from the beginning of hospitalization so that a patient-tailored discharge plan can be established and reflected in treatment. It is necessary to actively utilize nursing data containing comprehensive information [11,12].

Therefore, this study aimed to develop a readmission early prediction model utilizing nursing data, including physical, mental, and social information for high-risk discharge patients.

## Methods

### Study Design and Setting

Our article follows the guidelines of the Transparent Reporting of a Multivariable Prediction Model for Individual Prognosis (TRIPOD) consensus document. This study is a retrospective electronic health record (EHR) data analysis. This retrospective observational study used EHR data collected at a tertiary acute care hospital with over 2500 medical conditions in Seoul, Republic of Korea. To develop a readmission early prediction model for readmission of high-risk discharge patients, we employed a retrospective study design utilizing nursing data.

### Data Sources

We extracted medical record data of 12,977 patients at S Hospital in Seoul, Korea, for 25 months from January 2018 to January 2020. As a study to confirm the EHR data from the time of admission to the time of discharge of the patient, the information collected until January 31, 2021, was used to track readmission within 30 days. Data were requested from S University Hospital via the SOBIG data portal using subject inclusion criteria. The big data team extracted and received data from the first hospitalization of adult patients hospitalized for 6 major disease groups (chronic obstructive pulmonary disease, liver disease, diabetes mellitus, lung disease, heart failure, and chronic kidney disease) for 2 years. Hospital S is a high-level general hospital with about 2500 beds, with approximately 2000 to 3000 surgeries per month and more than 10,000 outpatient visits per day. We secured medical records of medical institutions where many patients are frequently hospitalized and readmitted and used them to develop a prediction model.

We used various types of EHR data from the participants, including during hospitalization and follow-up data within 30 days of discharge point. Analysis in this study included general, clinical, and nursing data recorded after the direct assessment of patients.

### Data Inclusion and Exclusion Criteria

The data inclusion criteria are as follows: patients were older than 18 years at admission. The disease group selection of the subjects was based on the most recent 2021 HIRA (Health Insurance Review and Assessment Service) data, and the discharged patients from the top 6 main diagnosis groups with high readmission rates were selected. One patient had only one record.

The data exclusion criteria are as follows: patients who died during hospitalization. Data resulting in death were excluded from discharge records.

### Data Preprocessing

#### Data Extraction

Of the initial 12,977 pieces of data, 388 duplicates were excluded, and 10,076 pieces of data were confirmed. Of these, 9536 medical records were analyzed, excluding data from 540 patients who died and data from 2513 patients with diagnoses who did not meet the inclusion criteria. Records with more than 65% missing values or records with missing values in categorical variables were deleted, and the final 9028 medical records were used to develop a prediction model.

To predict early readmissions at the same time as the patient’s admission, we built two models, Model 1 using the initial data up to the first day of admission and Model 2 using the data from the entire hospital stay to just before discharge, with different data extraction times.

#### Data Imputation

Category and continuous variables with missing values were handled differently. For categorical variables, we excluded data by deleting missing values, and for continuous variables, we had about 25% missing values for BMI and ward severity, and less than 5% missing values for all other variables. For BMI, the proportion of missing values was higher than for other variables due to the large number of cases where only weight was entered among the patient’s height and weight measurements. For ward severity, there were missing values due to the patient’s room change, but there was no difference in predictive power based on missing values when training the model. These missing values were imputed using KNN imputation (K=5). Finally, we constructed a readmission prediction model using 9028 data.

#### Data Imbalance

The observed readmission rate for all data used in this study was 16.5%. To solve the data imbalance problem when building readmission prediction models, we compared four sampling methods: ADASYN, under-sampling, SMOTE, and random over-sampling. The ADASYN method was ultimately selected among the four sampling methods to resolve the data imbalance issue because the ADASYN method showed the highest possible model performance results.

### Ethical Considerations

The ethics review committee of Severance Hospital approved this retrospective data analysis study (DRB approval number 4-2021-1334). Severance Medical Center’s guidelines for using EHRs require the data to be provided in Excel format with patient identification information removed. Data analysis was performed based on this data.

### Variables

EHR data were analyzed to predict patient readmissions. Readmissions, the primary outcome of interest in the study, were assessed as unplanned readmissions within 30 days [13]. The study aimed to determine a patient’s likelihood of readmission to develop an early discharge care plan. To determine the likelihood of patient readmission, we classified the target variable Y as yes (1) or no (0). Predictor variables (X) necessary to confirm the presence or absence of rehospitalization, which is the target variable of the patient, including sex, age, admission days, diagnosis group, number of ICD-10 codes, admission and discharge medications, state of consciousness, BMI, falls and pressure ulcer risk, vital signs, literacy, economic status, functional and emotional evaluation, number of nursing diagnosis records, presence of the caregiver, admission via the emergency room, health behavior habits (smoking, drinking, regular exercise, sleep disorder, and nutritional status), blood test values (levels of sodium, hemoglobin, potassium, aspartate aminotransferase, glucose, and creatinine; and white blood cell counts) were included in the analysis. Utilizing coefficient of variation (CV) values, Model 2 incorporated patient data from admission to discharge to capture data variability.

Among the various variables, the following are included as nursing data: falls and pressure ulcer risks, vital signs, literacy, economic status, functional and emotional evaluation, number of nursing diagnosis records, presence of the caregiver, health behavior habits (eg, smoking, drinking, regular exercise, sleep disorder, and nutritional status), and ward severity. The ward severity score is a score given by the nurse in charge of the ward to each patient by identifying the number of interventions in the areas of hygiene, nutrition, elimination, exercise and activity, education and counselling, emotional support, measurement and observation, communication and alertness, treatment and testing, medication, interdepartmental coordination of patient care, discharge, and power management. The modified Barthel index scale is an ordinal scale that measures an individual’s ability to complete activities of daily living. It consists of 10 items and has a total score of 100. However, the organization that collected the data evaluated 11 items by adding an item on the ability to use walking aids other than wheelchairs, and the total score was 105. All of these nursing data variables include information recorded by the nurse during the patient’s admission to the hospital to indicate the patient’s condition. We built a model to predict unexpected readmissions, divided into Model 1 and Model 2. Model 1 entered possible variables based on data from the first day of hospitalization, and Model 2 selected variables based on data from the entire period of hospitalization. All variables were selected based on prior literature and entered through stepwise feature selection [14,15].

Table 1 displays all the input variables according to Models 1 and 2.

**Table 1.** An overview of the input variables.

Variable name	Type	Value (range)	Model
**General data**			
Sex	Nominal	Male or Female	1, 2
Patient age	Ordinal	(19, 102)	1, 2
Diagnosis group	Nominal	0, 1, 2, 3, 4, 5	1, 2
Length of stay	Ordinal	(0, 532)	2
BMI	Ordinal	(10.63, 51.54)	1, 2
Emergency room visits	Nominal	Yes or No	1, 2
Admission to the intensive care unit	Nominal	Yes or No	2
**Clinical data**			
ICD-10 code	Ordinal	(1, 29)	1, 2
Medicine	Ordinal	(0, 37)	1
Discharge medication	Ordinal	(0, 33)	2
Number of surgeries	Ordinal	(0, 6)	2
Mental status	Nominal	Alert or Non-Alert	1, 2
SBP^a^	Ordinal	(48, 255)	1
SBP_CV^b^	Ordinal	(0, 0.31)	2
DBP^c^	Ordinal	(20, 156)	1
Heart rate	Ordinal	(22, 195)	1
Heart rate_CV	Ordinal	(0, 0.52)	2
Body temperature	Ordinal	(34.5, 40.8)	1
Body temperature_CV	Ordinal	(0, 0.03)	2
Respiration	Ordinal	(8, 52)	1
Saturation	Ordinal	(62, 100)	1
Whole blood test^d^	Ordinal	(0, 5)	1
Chemistry test^e^	Ordinal	(0, 5)	1
Electrolyte test^f^	Ordinal	(0, 5)	1
WBC_CV	Ordinal	(0, 1.75)	2
Hemoglobin_CV	Ordinal	(0, 0.62)	2
Na_CV	Ordinal	(0, 0.09)	2
AST_CV	Ordinal	(0, 3.28)	2
Glucose_CV	Ordinal	(0, 1.66)	2
K_CV	Ordinal	(0, 0.42)	2
Creatinine_CV	Ordinal	(0, 1.32)	2
**Nursing data**			
Number of nursing diagnosis	Ordinal	(0, 15)	1, 2
Ward severity	Ordinal	(9, 48)	1
Ward severity_CV	Ordinal	(0, 0.71)	2
Caregiver	Nominal	Yes or No	1, 2
Health behavior habits^g^	Ordinal	(0, 8)	1, 2
Fall risk assessment	Ordinal	(0, 20)	1
Fall risk assessment_CV	Ordinal	(0, 3.06)	2
Braden scale	Ordinal	(10, 26)	1
Braden scale_CV	Ordinal	(0, 0.33)	2
Literacy	Nominal	Adequate or Inadequate	1, 2
Financial problem	Nominal	Yes or No	1, 2
Emotional assessment	Ordinal	(0, 6)	1, 2
Modified Barthel index	Ordinal	(0, 105)	1, 2

a SBP: systolic blood pressure.

b CV: coefficient of variation.

c DBP: diastolic blood pressure.

d Whole blood test: The sum of the normal items of the white blood cell count, hemoglobin, hematocrit, platelet count, and lymphocyte tests among the complete blood count tests.

e Chemistry test: The sum of the normal items of blood urea nitrogen, creatinine, aspartate aminotransferase, alanine transaminase, and albumin tests among routine chemistry serum tests.

f Electrolyte test: The sum of the normal items of calcium, inorganic P, Na, K, and Cl tests among electrocyte serum tests.

g Health behavior habits: smoking history, drinking history, sleep disorder, regular exercise, and nutritional status.

### Machine Learning-Based Predictive Models

Machine learning is a field of artificial intelligence that studies the development of computer algorithms and techniques that automatically improve through experience [16]. Machine learning is divided into supervised learning and unsupervised learning and predicts outcomes through supervised learning. There are various types of machine learning. A representative example is a decision tree, a graph representing a selection and its result in the form of a tree. The nodes of the graph represent events or selections, and the edges of the graph represent decision rules or conditions. Each tree consists of nodes and branches. Each node represents an attribute of a group to be classified, and each branch represents a value that the node can take. It is an algorithm suitable for a rehospitalization prediction model that mainly predicts the presence or absence of rehospitalization. The boosting algorithm is a family of algorithms that transform weak learners into strong learners. Boosting is an ensemble learning technique used to reduce bias and variance. A weak learner is defined as a classifier, and a strong learner is a classifier that is randomly correlated with the actual classification. Many algorithms with high accuracy and predictive power are being developed in prediction algorithms. Bagging is applied where the accuracy and stability of machine learning algorithms must be increased, and it can be applied to classification and regression and helps reduce variance and handle overfitting. The random forest algorithm is an algorithm that creates a decision tree using this bagging method. It is an algorithm that can solve the overfitting problem that a single decision tree can have by creating various subdatasets through bootstrapping and having multiple decision trees learn each dataset and collate the results [6]. In this study, we used a supervised learning method through 6 algorithms that are considered most suitable for predicting readmission among machine learning algorithms: logistic regression, decision tree, random forest, CatBoost, XGBoost (extreme gradient boosting), and multilayer perceptron, and developed a readmission prediction model for high-risk patients. The dataset was split into 60% for training, 20% for validation, and 20% for testing, with all models trained 5 times and model performance evaluated using layered 5-fold cross-validation. We performed a grid search to find the most suitable hyperparameters for the model while training and evaluating the model, and then performed model fine tuning.

According to the data inclusion and exclusion criteria and missing value processing, data from 9028 patients were available for analysis. We constructed unexpected readmission prediction models by dividing them into Model 1 and Model 2. Model 1 implemented an early readmission prediction model based on data from the first day of hospitalization to predict readmission early. Model 2 implemented a model to supplement data that should have been included in Model 1 based on all the data. All machine learning–based predictive model development processes were conducted with the participation of artificial intelligence experts, who conducted the procedures together, provided advice, and reviewed them. The analysis in this study was performed using Python Language Reference, version 3.11.3. (Python Software Foundation).

### Evaluation of Prediction Performance

After implementing 6 models, we selected the final model and evaluated each model’s confusion matrix, accuracy, precision, recall, F1-score, and area under the receiver operating characteristic (AUROC) curve.

We compared techniques using performance measures of different models. Confusion matrices were used to determine accuracy, recall, precision, and F1-score. True positive, true negative, false positive, and false negative were used to determine the performance of each model. True positive refers to cases where the model predicted yes, and the patient was actually readmitted. In contrast, true negative refers to cases where the model predicted no, and the patient was not readmitted. Conversely, false positive refers to cases where the model predicted yes, but the patient was not actually readmitted, and false negative refers to cases where the model predicted no, but the patient was not actually readmitted.

## Results

**Figure 1.**
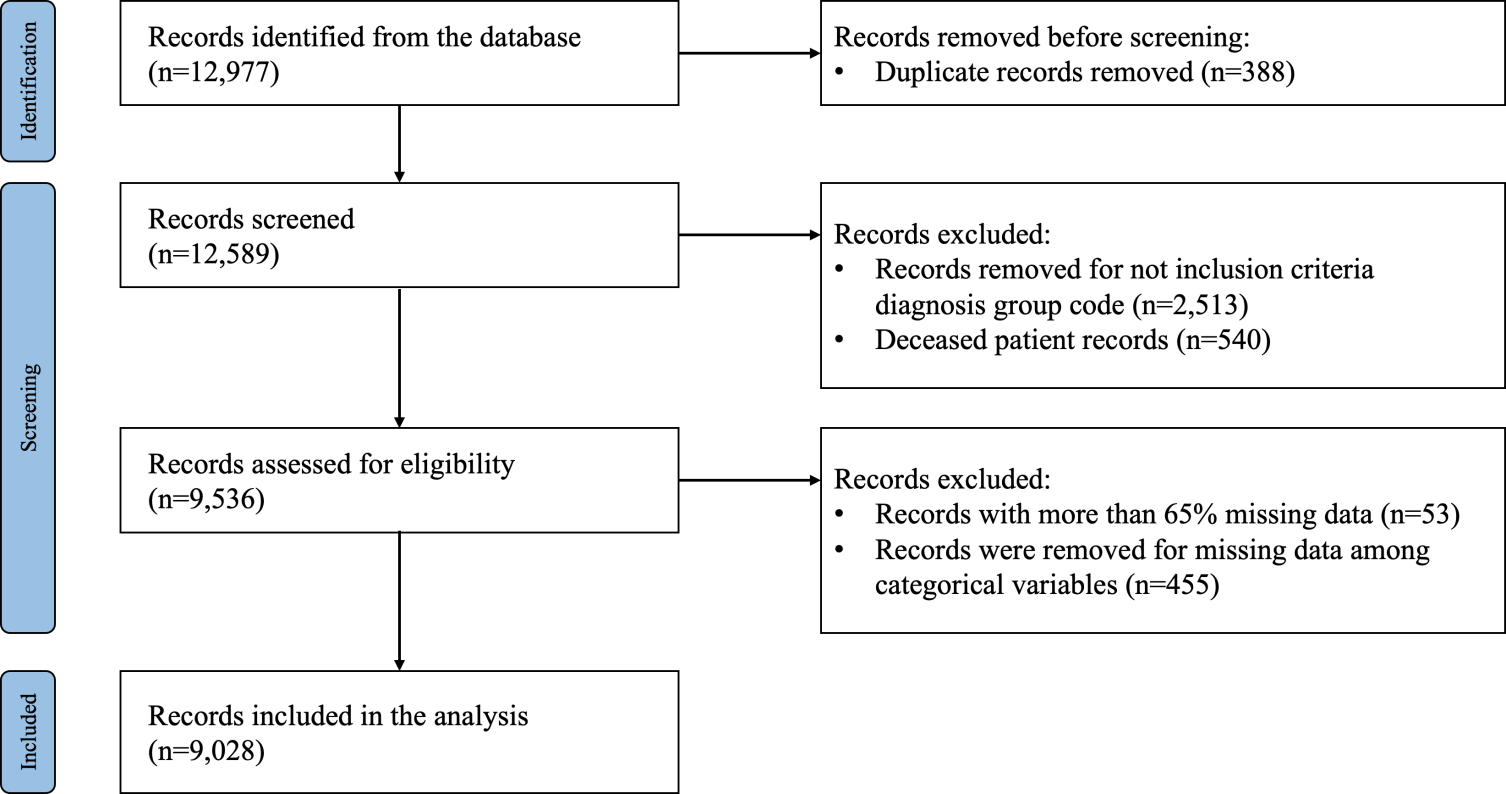
Flow chart showing data extraction.

Figure 1 shows the flow of data extraction. Of the initial 12,589 data, we analyzed a final of 9536 medical records, excluding 2513 duplicates and 540 deceased patient data. Of the 9028 participants, 1493 experienced an unplanned readmission within 30 days; the rate of unplanned readmissions within 30 days was 16.5%. Table 2 shows the essential characteristics of the study participants. There were 3186 (35.29%) patients under the age of 60 years, 1194 (13.23%) patients over the age of 80 years, and 4648 (51.48%) patients between the ages of 60 and 80 years. In terms of sex, there were 3309 (36.65%) men and 5719 (63.35%) women in the study, and by diagnosis, 4725 (52.34%) patients had liver disease, followed by 1612 (17.86%) patients with pulmonary disease, 1309 (14.50%) with chronic kidney disease, 1004 (11.12%) with heart failure, 279 (3.09%) with diabetes, and 99 (1.10%) with chronic obstructive pulmonary disease. A total of 3237 (35.86%) patients were admitted unplanned via the emergency department, while 5791 (64.14%) were planned via the outpatient department.

**Table 2.** Demographic information of the dataset.

Patient characteristics	n (%) (N=9028)
Sex	
Male	3312 (36.69)
Female	5716 (63.31)
Age (years)	
<60	3185 (35.28)
60-79	4650 (51.51)
≥80	1193 (13.21)
Diagnosis group	
COPD^a^	99 (1.10)
Liver disease	4728 (52.36)
Diabetes mellitus	278 (3.08)
Pulmonary disease	1612 (17.86)
Heart failure	1004 (11.12)
CKD^b^	1307 (14.48)
Admission route	
Emergency room	3238 (35.87)
Outpatient	5790 (64.13)
Readmission	
Yes	1492 (16.53)
No	7536 (83.47)

a COPD: chronic obstructive pulmonary disease

b CKD: chronic kidney disease

In this study, we developed 2 prediction models to enable the early prediction of high-risk patient readmissions when admitted to the hospital: one using early hospitalization data and the other using complete data that can compensate for the initial lack of information. Model 1 was a high-risk patient readmission prediction model developed using initial data up to the first day of hospitalization. Model 2 was a prediction model developed based on complete patient hospitalization data. The performance evaluation results of the 5-fold cross-validation test of the 6 candidate model algorithms in Model 1 and Model 2 are shown in Table 3.

**Table 3.** Comparison of the performance of the predictive models of Model 1 and Model 2.

Algorithm	Set	Accuracy	Precision	Recall	F1-score	AUROC^a^ curve
Model 1						
LR^b^	Train	0.59	0.58	0.62	0.60	0.61
	Test	0.83	0.20	0	0	0.60
DT^c^	Train	0.84	0.98	0.70	0.82	0.88
	Test	0.83	0.21	0	0	0.55
RF^d^	Train	0.89	0.99	0.78	0.87	0.95
	Test	0.79	0.25	0.57	0.33	0.62
CatBoost	Train	0.89	0.99	0.78	0.88	0.93
	Test	0.83	0.28	0	0	0.62
XGB^e^	Train	0.85	0.94	0.76	0.84	0.92
	Test	0.83	0.25	0	0	0.60
MLP^f^	Train	0.62	0.68	0.55	0.55	0.72
	Test	0.83	0.21	0.01	0.02	0.60
Model 2						
LR	Train	0.60	0.59	0.61	0.60	0.63
	Test	0.83	0	0	0	0.62
DT	Train	0.83	0.93	0.72	0.81	0.88
	Test	0.83	0	0	0	0.61
RF	Train	0.83	0.95	0.70	0.80	0.90
	Test	0.59	0.22	0.59	0.10	0.63
CatBoost	Train	0.86	0.98	0.73	0.84	0.92
	Test	0.83	0	0	0	0.64
XGB	Train	0.99	1	0.99	0.99	1
	Test	0.82	0.24	0.05	0.08	0.62
MLP	Train	0.76	0.75	0.78	0.76	0.84
	Test	0.83	0.45	0.03	0.06	0.64

a AUROC: area under a receiver operating characteristic.

b LR: logistic regression.

c DT: decision tree.

d RF: Random forest.

e XGBoost: extreme gradient boosting.

f MLP: multilayer perceptron.

**Figure 2.**
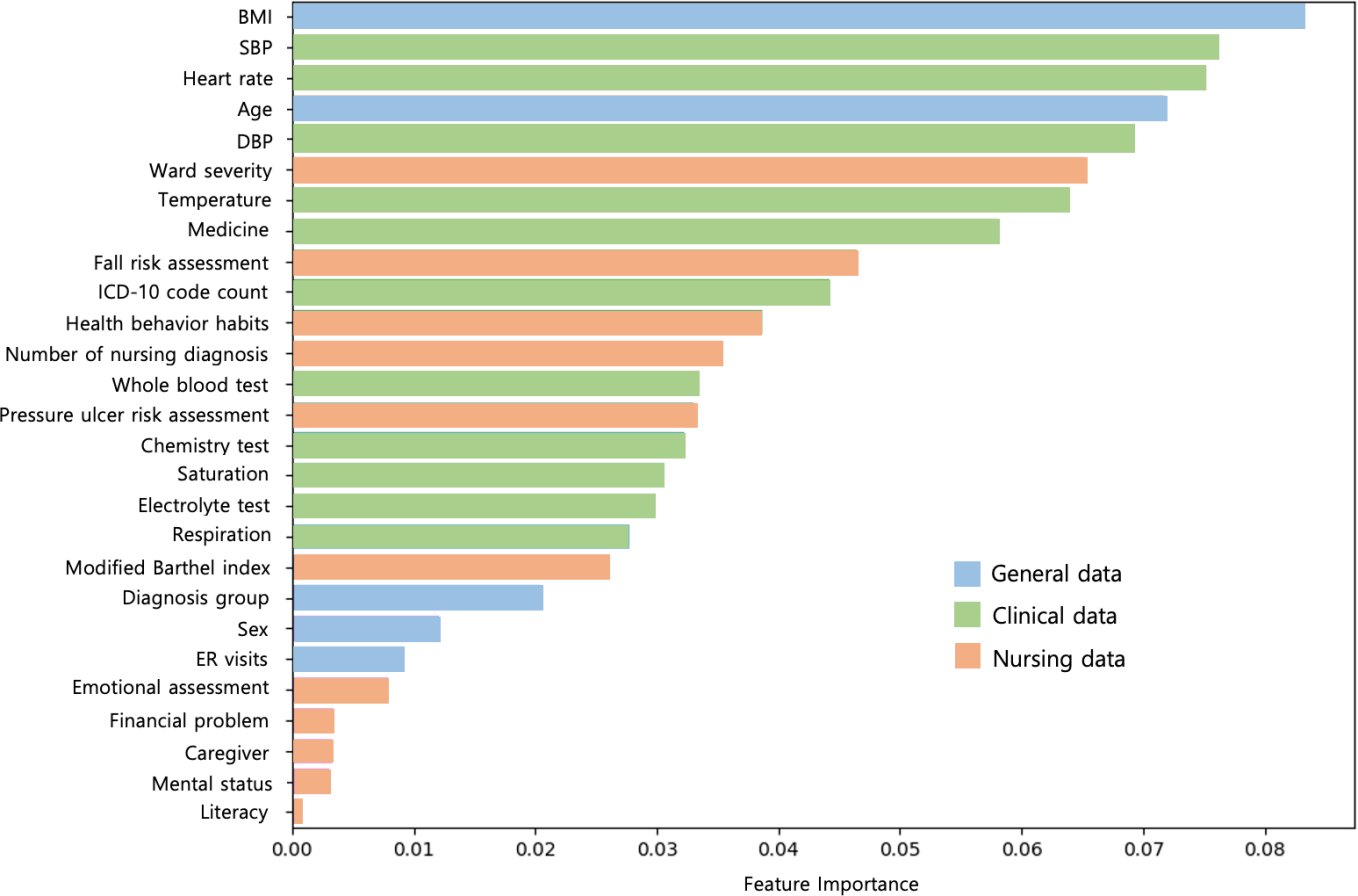
Feature importance of the random forest model in Model 1. SBP: systolic blood pressure; DBP: diastolic blood pressure; ER: emergency room.

**Figure 3.**
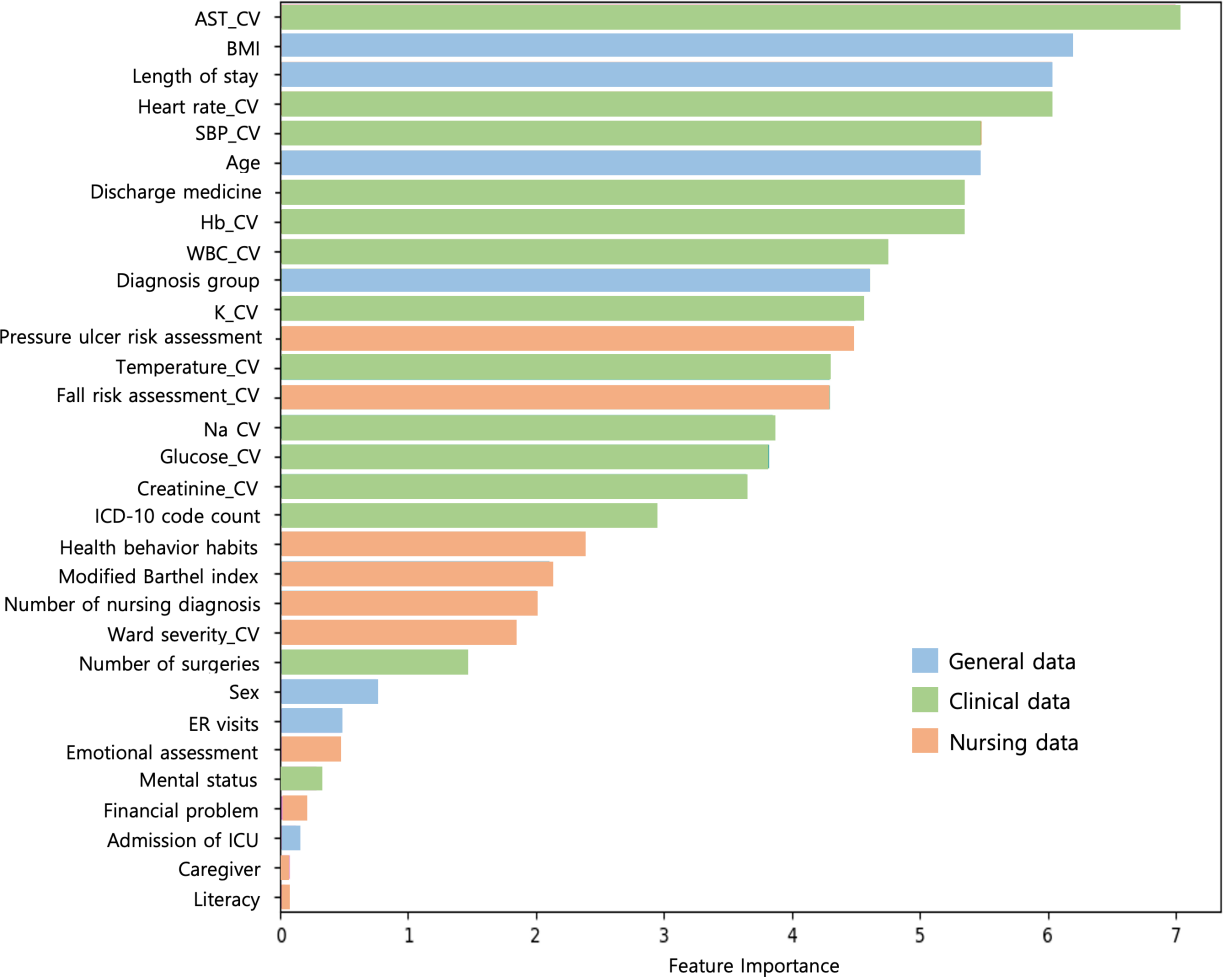
Feature importance of the CatBoost model in Model 2. AST: aspartate transaminase; CV: coefficient of variation; SBP: systolic blood pressure; Hb: hemoglobin; WBC: white blood cell; ER: emergency room; ICU: intensive care unit.

For Model 1, the random forest model had the highest AUROC curve of 0.62 compared to all other models, while the decision tree model performed the worst with an AUROC curve of 0.55. The feature importance of the best-performing random forest model in Model 1 is shown in Figure 2. In Model 2, the CatBoost model had the highest AUROC curve of 0.64 compared to all other models, while the decision tree model had the lowest performance with an AUROC curve of 0.61. The feature importance of the best-performing CatBoost model in Model 2 is shown in Figure 3. Ultimately, the best-performing prediction models were the random forest model for Model 1 and the CatBoost model for Model 2. Although the random forest model and CatBoost model have the same AUROC curve value, when comparing the performance values of all models including the training data, the random forest model was not overfitted and performed evenly well, so after statistical discussion with AI experts, it was selected as the Model 1 final model.

As a result of analyzing the feature importance of Model 1 and Model 2, the three variables of BMI, systolic blood pressure, and age were commonly found to be important predictors of readmission risk. According to Figure 2, Model 1, which enabled the early prediction of readmission, had a higher proportion of nursing data among the important predictors than Model 2. In Model 1, constructed using early hospitalization data, 45% of the nursing data variables (ward severity, fall risk assessment, health behavior habits, number of nursing diagnoses, and pressure ulcer risk assessment) were ranked within the top 50% of important variables. This finding suggests the potential significance of incorporating nursing data in early readmission prediction models.

## Discussion

### Principal Findings and Comparison With Previous Works

In predicting unplanned readmission within 30 days using data from 9028 high-risk discharged patients, machine learning methods (random forest and CatBoost models) showed better predictive power than traditional logistic regression methods, similar to other previous studies [17,18]. While previous studies have focused on developing a prediction model for a specific condition for a specific group of patients with a specific diagnosis [19], this study is significant in that it is a prediction model development study that focuses on the generalization process of predicting unplanned readmissions within 30 days by incorporating the top 6 diagnosis groups of high-risk discharged patients suggested by the Health Insurance Review and Assessment Service to predict the potential of readmission.

Many interventions aimed at reducing patients’ unplanned readmissions are often initiated just before or after inpatient discharge, which has little impact on improving the inpatient quality of care [20]. Providing early interventions related to readmissions during hospitalization with early discharge planning can reduce readmissions [21]. However, most readmission prediction models are built on posthospitalization data, often including information about the patient’s physical and diagnostic tests. Because clinically essential variables such as the length of stay, diagnosis codes, laboratory test results, and medications are usually entered just before discharge, readmission prediction models are primarily built on patient data immediately before or after discharge. The most widely used measures of readmission risk in US health care settings are the HOSPITAL score [22] and the LACE index [23], but both have the limitation that they are only available at the end of a hospital stay. When creating a prediction model that includes all variables, its prediction performance is good. However, it needs to be applied for more time to actual patients, making it challenging to support effective clinical decision-making. In this study, to overcome these limitations, we implemented two patient readmission prediction models, Model 1 and Model 2, to predict the readmission rate from the time of hospitalization and plan discharge care. Model 1, implemented based on the patient’s initial hospitalization data, and Model 2, implemented by adding variables that can complement Model 1, can help clinical decision-making by predicting the patient’s readmission rate earlier. Model 1, which utilized data from the first day of hospitalization, had an AUROC curve of 0.62 in our study. Although this performance is not exceptionally high, it is consistent with most previous studies that developed readmission prediction models using initial hospitalization data, which reported results of less than an AUROC curve of 0.75 [5,17,24,25]. Importantly, it enables the proactive identification of high-risk patients and the provision of timely and preventive therapeutic interventions early in their hospital stay.

Unplanned readmissions cause a heavy burden on individuals and society through unnecessary health care costs and resource use, and readmission management has been recognized as an essential issue in improving patient quality of care [26]. To reduce the cost of unnecessary readmissions and improve the quality of care for patients, the Korea Health Insurance Review and Assessment Service conducts a national risk-based readmission cost appropriateness assessment, and the US government has implemented the Hospital Readmission Reduction Program (HRRP) since 2012 to encourage the reduction of hospital readmissions by involving patients and caregivers in discharge planning [27]. However, limited health care resources make it challenging to provide discharge interventions to all patients, and screening high-risk patients for early discharge planning is essential. Evidence suggests that focusing interventions on high-risk patients can reduce the risk of hospital readmission within 30 days by 11‐28% [28-30], and it is essential to utilize nursing data that include holistic information from the patient’s initial hospitalization to develop a preventive care plan for high-risk patients. A previous study found the potential importance of utilizing nursing variables to identify risk factors for readmission [31]. The BMI variable at the top of the feature importance of Model 1 is the same as the result that it was highly related to the patient’s obesity level, cardiovascular problems, and readmission, similar to the previous study [32]. This study is significant in that it proved that BMI is the most simple and important screening variable related to the patient’s readmission in the early stage of hospitalization. In the future, it is necessary to recognize the importance of BMI screening and collect information related to the probability of early patient readmission [32].

Information collected directly about patients’ physical, mental, and social aspects by nurses, who interact most closely with patients in the hospital and provide care, is essential in predictive models for the early identification of high-risk discharges. In this study, we developed an early prediction model for unplanned readmissions using nursing data from patients in the early stage of hospitalization. In particular, we found that many nursing data variables were included in the high rankings among the important predictors in Model 1 developed using early hospitalization data. The high representation of nursing variables among the most influential predictors underscores the potential value of integrating nursing-specific information into risk assessment tools for hospital readmission. Further investigation into the specific contributions of these nursing variables may provide valuable insights for enhancing the accuracy and clinical utility of early readmission prediction models. Although most predictions of high-risk discharged patients are based on data at discharge, identifying high-risk patients should ideally be performed early enough to allow for therapeutic interventions during hospitalization, which was attempted in this study.

### Limitations

This study has several limitations. First, our prediction model, designed for adults aged over 18 years, does not apply to pediatric patients aged less than 18 years. Second, the model’s development was based on data from a single hospital in a specific region. Consequently, its applicability to readmission scenarios in different health care settings or countries may be limited. Furthermore, tracking patient readmissions poses a challenge, especially in cases where patients admitted to our hospital could have subsequent readmissions to different facilities. This potential for cross-hospital readmissions might not be fully captured in our model.

Additionally, as this was a retrospective study utilizing deidentified data, it limited our ability to validate the relationships between our findings and factors identified in other studies. Finally, there were limitations in implementing perfectly standardized data, as the data were manually entered as part of the medical and nursing data.

### Clinical Implications

Our readmission prediction model can be used to predict and continuously monitor a patient’s risk of readmission during the entire hospital stay. It can be used as an early screening tool to assess the risk associated with a patient’s readmission.

### Conclusions

This study developed two prediction models, Model 1 using initial hospitalization data and Model 2 reflecting the variability of variables from admission to discharge, for the early prediction of unplanned readmission within 30 days in high-risk discharged patients. Model 1 showed the best performance of the random forest model, with an AUROC curve of 0.95 for the training data and 0.62 for the test data. For Model 2, the CatBoost model performed the best, with an AUROC curve of 0.92 for the training data and an AUROC curve of 0.64 for the test data. The Model 1 and Model 2 predictive models allow us to predict the likelihood of readmission from the beginning of a patient’s hospitalization and proactively plan for early discharge. In addition, this study identified various significant predictors of unplanned readmission and identified changes in patients over time. The predictive model was developed and validated using data from 9028 discharged patients and can be applied to adult patients who represent the high-risk discharge population in tertiary care hospitals. However, further research and experimentation are necessary to implement the developed predictive models in clinical practice within an EHR environment for predicting early readmission and monitoring readmission risk. In particular, additional studies must be conducted to address the limitations of this monocentric study, consider the characteristics of medical data with numerous missing values, and validate the data sources for each variable.

Based on this study, future development of a systematic clinical decision support system that utilizes machine learning–based readmission prediction models with nursing data could enable real-time early risk prediction of patient readmission. This approach would facilitate the provision of preventive discharge services for high-risk patients and improve the quality of health care services.

## Abbreviations

AUROC: area under a receiver operating characteristic
CV: coefficient of variation
EHR: electronic health record
F1: harmonic mean of precision and recall
HRRP: Hospital Readmission Reduction Program
